# Factors and key problems influencing insured’s poor perceptions of convenience of basic medical insurance: a mixed methods research of a northern city in China

**DOI:** 10.1186/s12889-023-15993-1

**Published:** 2023-06-05

**Authors:** Peng Wang, Lixia Cheng, Ye Li, Yuchao Zhang, Weiqi Huang, Shuyi Li, Zhizhen Wang, Linghan Shan, Mingli Jiao, Qunhong Wu

**Affiliations:** 1 Department of Social Medicine, School of Health Management, Harbin Medical University, No.157 Baojian Road, Nangang District, Harbin, 150081 China; 2 School of Public Health, Harbin Medical University, Harbin, 150081 China; 3 Department of Biostatistics, School of Public Health, Harbin Medical University, Harbin, 150081 China; 4 Research Center of Health Policy and Management, School of Health Management, Harbin Medical University, No.157 Baojian Road, Nangang District, Harbin, 150081 China

**Keywords:** Basic medical insurance system, Insured, Perceptions of convenience, Basic medical insurance policy, Public cognition, Mixed methods research

## Abstract

**Background:**

This study aimed to explore the factors that affect insured’s perceptions of convenience of the basic medical insurance (PCBMI) in Harbin, China and to diagnose the key problems to further propose corresponding measures. The findings provide evidence-based support for the reform of convenience of the basic medical insurance system (BMIS) and the cultivation of public literacy.

**Methods:**

We adopted a mixed methods design composing a multivariate regression model using the data from a cross-sectional questionnaire survey (*n* = 1045) of residents who were enrolled for BMIS in Harbin to identify the factors influencing the PCBMI. A quota sampling method was further adopted. Semi-structured interviews were then conducted with 30 important information providers selected by convenience sampling. Interpretative phenomenological analysis was employed to summarize and analyze the key problems.

**Results:**

Overall, approximately 51% of respondents reported poor PCBMI. The logistic regression model showed that insured without outpatient experience within two weeks (OR = 2.522, 95% CI = 1.267–5.024), had poorer levels of understanding of basic medical insurance information (OR = 2.336, 95% CI = 1.612–3.386), lived in rural areas (OR = 1.819, 95% CI = 1.036–3.195), had low levels of annual out-of-pocket medical expenses (OR = 1.488, 95% CI = 1.129–1.961), and were more likely to give the PCBMI a worse evaluation than their counterparts. The results of the qualitative analysis showed that the key problem areas of the PCBMI were the design of the BMIS, the cognitive biases of the insured, publicity information about the BMIS, and the health system environment.

**Conclusions:**

This study found that in addition to the design of BMIS, the cognition of the insured, the BMIS information publicity and the health system environment are also the key problems hindering PCBMI. While optimizing system design and implementation, Chinese policymakers need to focus on the insured with low PCBMI characteristics. Moreover, it is necessary to focus on exploring effective BMIS information publicity methods, supporting public policy literacy and improving the health system environment.

**Supplementary Information:**

The online version contains supplementary material available at 10.1186/s12889-023-15993-1.

## Background

After the World Health Organization proposed the concept and strategic goal of universal health coverage [1, 2], the Chinese government implemented the reform of universal basic medical insurance system (BMIS) in 2009, and made remarkable achievements in terms of population coverage [3]. As the reform entered the deepening stage, China’s focus on the development of medical insurance has gradually shifted from the quantity of coverage to the depth of service quality [4]. Perceptions of the convenience of basic medical insurance (PCBMI) is an important part of the reform [5]. It reflects the rationality and coordination of the arrangement of various elements of the BMIS, as well as the literacy of the insured about basic medical insurance service utilization. Poor PCBMI not only affects whether the insured can fully obtain timeous medical economic compensation, but also becomes an obstacle to the use of medical services [6]. Moreover, the gap in convenience expectations caused by the insured’s incorrect cognition can easily induce violations, solidify incorrect habits, and intensify doctor-patient conflicts if it cannot be timeously investigated and reasonably guided [7–9].

During the “Fourteenth Five-Year Plan” period, China proposed requirements to improve the efficiency and shorten the time costs of basic medical insurance. Additionally, government documents have clearly pointed out specific development indicators, such as the cross provincial direct settlement rate of hospitalization expenses, online processing rate, and artificial windows processing rate of the basic medical insurance business [10]. While a series of convenience reforms have been implemented at all levels of medical insurance institutions in China [11, 12], there remains a significant gap between the convenience of basic medical insurance services and the insured’s expectations.

Previous studies have shown that the insured are not satisfied with the convenience of basic medical insurance. For instance, 54% of 1,277 insured in four cities in China thought they had poor portability of basic medical insurance [13]. Another survey on cross regional medical treatment showed that 31.9% of 1,672 patients encountered barriers to the use of basic medical insurance. For example, the procedures for reporting cross regional health-seeking behavior to the local administration system of medical insurance were complex, and outpatient service expenses could not be reimbursed [14]. Moreover, there are also a series of problems in other aspects in terms of convenience, such as difficulties in transferring and connecting different basic medical insurance systems [15], complex reimbursement procedures [16], reimbursement delays [17], and the long journeys to designated hospitals for basic medical insurance [18].

To improve the convenience of basic medical insurance, new requirements for medical insurance related research have been proposed, which requires empirical research that can systematically evaluate and reflect the insured’s perceptions about the convenience of basic medical insurance. However, thus far, the evaluation of the effect of the basic medical insurance convenience reform has mostly focused on the performance of the policy output side, which is usually measured by some objective indicators, such as effective person-times of direct settlement for medical treatment in non-residential places [19, 20] and online processing times of basic medical insurance business [21]. There is a lack of research on the evaluation of real perceptions of the convenience reform from the insured’s side. The evaluation with the policy output subject as the core probably covers up the actual difficulties of the insured, and also ignores that good policy effects are the product of the interaction between the government and the public in cognition and action [22]. Second, previous studies only took the perception of convenience as an independent variable and focused on single content [13]. They did not adequately conduct multi-angle research on convenience as a dependent variable. Third, some studies on the perceptions of convenience of basic medical insurance often adopted quantitative research, mainly statistical analysis of the influencing factors [17]. However, quantitative research makes it difficult to measure and conduct an in-depth analysis of unquantifiable problems [23]. Since the insured’s perceptions of convenience are subjective feelings, qualitative research is required to identify hidden problems and understand the formation and development of the perceptions of convenience.

At present, the effects of the basic medical insurance convenience reform are not clear due to grossly insufficient research on the PCBMI of the insured. As the core actors and beneficiaries of the use of basic medical insurance, how do the insured perceive the convenience reform effect of basic medical insurance? What factors affect their perceptions? What are the key challenges and problems? The answers to these questions are crucial for evaluating the current convenience reform of basic medical insurance and improving the perceptions of benefits of the insured. Therefore, this study, based on the perspectives of the insured, explored their perceptions of the convenience and performance of basic medical insurance from influencing factors and key problems using a mixed research method. It provides new insights for policy makers to further understand and optimize the practical problems of basic medical insurance convenience reform. In addition, it is expected that the research findings can provide a reference to promote convenience reform in other countries where the effectiveness of the arrangement of various elements of basic medical insurance remains a challenge.

## Methods

### Research design

The study was conducted in Harbin, China, which is the capital of Heilongjiang Province located in Northeast China. The city had a population of 9.885 million people and a per capita GDP of 53,517 CNY in 2021, which is comparatively lower than other provincial capitals in China. In general, the basic policies and regulations of China's BMIS are formulated by the national government, and local governments have some flexibility to make appropriate adjustments according to their specific circumstances while adhering to the fundamental principles of the system. National policies mainly govern the basic structure of the BMIS, necessary reforms, and minimum guarantee standards. The capable local governments are permitted to conduct pilot explorations in line with the national trend of BMIS reform. However, only a small percentage of regions in China have piloted advanced system reforms. The arrangement and reform of the BMIS in Harbin, Heilongjiang Province is consistent with most regions in China and essentially mirrors national basic policies and regulations, which can, to a certain extent, reflect some common phenomena and problems of regions similar to Harbin.

A mixed research approach was used in this study. Quantitative research was conducted to identify the characteristics of the insured with poor PCBMI and determine the influence factors, and qualitative research was conducted to explore the key problems of poor PCBMI from the perspective of the insured. According to the research results, the study proposed countermeasures and suggestions.

### Quantitative research

**Fig. 1:**
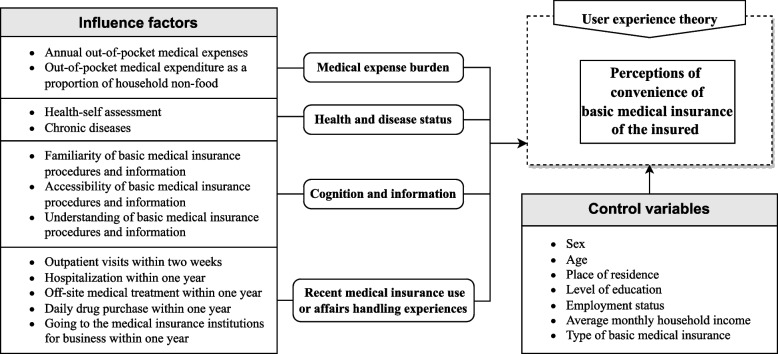
Analytical framework of insured’s PCBMI

An analytical framework was established based on a theory and some research findings (Fig. 1). User experience theory (UE) showed that the products used should be evaluated from the perspective of users’ perceptions and emotions [24]. Additionally, many researchers showed that “medical expense burden” was associated with the convenience of basic medical insurance reimbursement [25]. Ma et al. and Li et al. proposed that “health status” and the “policy information awareness” of the insured were the important aspects affecting their assessments of the convenience of BMIS [6, 26]. Further, Sanogo et al. viewed “insured experience” as fundamental for the insured to evaluate the convenience of BMIS services [27].

Based on this analytical framework, a six-part questionnaire was developed which gathered data on the insured’s (1) demographic and socioeconomic characteristics, (2) health and disease status, (3) medical expense burden, (4) mastery and accessibility of basic medical insurance information, (5) basic medical insurance use experience, and (6) the PCBMI of the insured.

The quantitative survey was conducted from December 2020 to February 2021. The researchers invited residents aged 18 and above without cognitive impairment to complete a cross-sectional survey. Considering recruiting a representative sample for Harbin, we used the quota sampling method to ensure the sample represented Harbin’s general population in terms of sex, age, education level, employment status, two-week outpatient visits, hospitalization in the last year, and overall chronic disease incidences. Quotas for these criteria were set based on the Heilongjiang Statistical Yearbook (2020), China Health Statistical Yearbook (2019), China National Health and Nutrition Big Data Report (2018) [28, 30], and our past research experience. All respondents agreed to participate in the questionnaire before the survey. The sample size was estimated based on the need for logistic regression analysis: ten times more than the number of independent variables [31].

The questionnaires were conducted through face-to-face surveys under the guidance of professional staff who were specially trained. As this survey was conducted during the outbreak of COVID-19, there was a decrease in the utilization of outpatient and inpatient services by the insured compared to previous periods. A total of 1063 residents who enrolled for BMIS completed the questionnaire. We carefully tested the quality of the returned questionnaires to eliminate those that were incomplete or had logical errors or took less than 300 s to complete. The final sample size was 1045, with an effective recovery rate of 98.31%.

To ensure the quality of the collected data, we conducted a thorough analysis of the questionnaire's reliability and validity. Reliability can reflect the stability and concentration of data [32].We tested the reliability of the scales in this study. The Cronbach’s alpha of this study is 0.926 (> 0.9), indicating good reliability and consistency of the data [33]. Validity refers to the fact that a tool measures exactly what it proposes to measure [32]. The validity analysis of the scale data was performed by the factor analysis method combined with content validity. According to the result of validity analysis, the value of Kaiser–Meyer–Olkin (KMO) was 0.755, the factor load coefficient of the corresponding factors of the items was larger than 0.5, the cumulative variance contribution rate was 62.05%, the publicity was greater than 0.5, and there was no serious deviation in the corresponding relationship between items and factors. Statistical analysis of the content validity showed that the Scale Content Validity Index/universal agreement (SCVI/ UA) was 0.8, Scale Content Validity Index/average (SCVI/ave) was 0.97, and all item-level content validity indices (ICVIs) were all greater than 0.78. The above results proved that the questionnaire had relatively good validity [34].

A multiple logistic regression analysis was conducted to determine the factors influencing the insured’s PCBMI.

### Dependent variable

This study measured PCBMI with a total of 10 items divided into three dimensions: daily affairs procedure (convenience of participating in insurance procedures, procedures for payment of insurance premiums, and basic medical insurance transfer and connection), actual utilization (convenience of outpatient and emergency reimbursement procedures, hospitalization reimbursement procedures, off-site medical reimbursement procedures, recorded procedures of off-site medicals, and daily drug purchase reimbursement procedures) and spatial accessibility (geographic distribution convenience of designated medical institutions and designated pharmacies). Each item was scored using a 5-point Likert scale (1 = completely disagree, 2 = disagree, 3 = neither disagree nor agree, 4 = agree, 5 = completely agree). To facilitate multiple logistic regression analysis, the median method was used to classify the total scores of the 10 items into binary classification: 1 = poor (≤ 34) and 0 = good (> 34).

#### Health and disease status

Survey respondents were requested to answer the following questions: “Out of 100, how would you rate your current health status?” and “Please select the top three major chronic diseases you have been diagnosed with according to the options in the questionnaire.” In the regression modelling, the median method was used to classify the health self-ratings into two categories: 1 = poor (≤ 85) and 0 = good (> 85). The chronic disease status was divided into two categories: 1 = with chronic disease and 0 = no chronic disease of any kind.

#### Medical expense burden

Survey respondents were requested to answer the following questions: “What were your family’s annual out-of-pocket medical expenses in the previous year?” and “What were your family’s annual non-food expenses in the previous year?” In the regression modelling, the median method was used to classify annual family out-of-pocket medical expenses into two categories: 1 = high (> 3000) and 0 = low (≤ 3000). The proportion of family’s out-of-pocket medical expenses to family’s non-food expenses was divided into two categories by the median method: 1 = high (> 12%) and 0 = low (≤ 12%).

#### Cognition and information

Respondents were asked to use a 5-point Likert scoring method (1 = completely disagree, 2 = disagree, 3 = neither disagree nor agree, 4 = agree, 5 = completely agree) to answer the following questions: “Do you agree that you have a good familiarity, accessibility, and understanding of basic medical insurance procedures and information?” In the regression model, the insured’s responses were divided into two categories: 1 = poor (including complete disagree, disagree, and neither disagree nor agree) and 0 = good (including agree and completely agree).

#### Recent basic medical insurance use or affairs handling experience

Survey respondents were requested to answer the following question: “Have you experienced any of the following: outpatient visits within two weeks, hospitalization within one year, preventive care within one year, daily drug purchases, visiting basic medical insurance institutions for business within one year, and receiving off-site medical treatment within one year?” In the regression modelling, participants’ responses were divided into two categories: 1 = have experience and 0 = no experience.

### Control variables

In the statistical analysis, we controlled the confounding effect of the socio-demographic characteristics (sex, age, place of residence, level of education, employment status, average monthly household income, type of BMIS) of the survey respondents.

### Data analysis

Data were analyzed using SPSS version 25.0. First, the Chi-square test or Fisher’s exact test were used to determine the relationship between the PCBMI and each independent variable. We then constructed two models. The first model included all the variables (Hosmer–Lemeshow test, ^*2*^ = 14.376, *P* = 0.072) (Supplementary file 1), the second model only included statistically significant variables (*P* < 0.05) in the Chi-square test or Fisher’s exact test (Hosmer–Lemeshow test, ^*2*^ = 11.420, *P* = 0.179). From a statistical point of view, the two modeling approaches generated consistent results, and the second model showed a better fit and slightly different odds ratios (ORs) compared to the first one. Therefore, we only show the results of the second model in this study.

### Qualitative research

Semi-structured interviews were conducted to gather information on the main problems related to the insured’s PCBMI. The following questions were asked: “How did you perceive the convenience of basic medical insurance in the following aspects: procedures for participation, transfer and continuation, medical expense reimbursement, daily drug purchase, the reasonableness of the geographical distribution of designated medical insurance institutions and designated pharmacies, and the impact of COVID-19 on the convenience of basic medical insurance?” We also gathered the demographic and socioeconomic characteristics of the respondents.

The interviews were conducted from February 2021 to April 2021. A convenient sampling method was used to select insured or their families who had recent basic medical insurance use experiences, and were deeply impressed and had the ability to express themselves clearly. After explaining the purpose and procedure of the interview, the researcher obtained the oral informed consent of the interviewees. Considering the serious development of the pandemic situation, the interviewers conducted telephone interviews and recorded them according to the interview outline. Each interview lasted about 30–60 min and 30 people were interviewed; the number relied on the degree of information saturation.

Using Microsoft Word and Excel, two investigators were selected to code each transcript together, and the discrepancies were reconciled through group discussions until a consensus was reached. The audio recordings were first transcribed, and then the interview materials were analyzed using interpretative phenomenological analysis (IPA) [35]. The main steps were as follows: 1) Read the transcription repeatedly, 2) Preliminary notes and coding, 3) Propose primary topics, 4) Find the connection between primary themes and form more advanced themes, 5) produce the report.

## Results

### Respondents’ characteristics and PCBMI

Among the 1,045 respondents, 50.9% were female, 65.2% were aged between 30 and 59, 92.4% lived in urban areas, 61.9% had a high school education or above, and 59.5% were employed. Further, 52.3% of the respondents participated in UEBMI.

Overall, approximately 51% of the insured had poor PCBMI. The Chi-square tests revealed that the poor PCBMI were associated with socio-demographic characteristics, medical expense burden, cognition and information, and recent basic medical insurance use experiences. The respondents who live in rural areas, have lower levels of education, have lower average monthly household incomes, and participate in urban and rural resident basic medical insurance have poorer PCBMI. Those who have lower annual out-of-pocket medical expenses are more likely to express poorer PCBMI. Moreover, Those who have poorer familiarity, accessibility, and understanding of BMIS information have a higher percentage of poor PCBMI. Those respondents who have no experience in outpatient visits within two weeks, hospitalization within one year, or daily drug purchases within one year also exhibit poorer PCBMI (Table 1).

**Table 1:** Characteristics of respondents and overall PCBMI (*n* = 1045)

	**n(%)**	**Poor PCBMI(%)**	**Good PCBMI(%)**	*χ*^*2*^	***P*-value**
**Social demography**					
**Sex**				2.254	0.133
Male	513 (49.1)	251 (48.9)	262 (51.1)		
Female	532 (50.9)	285 (53.6)	247 (46.4)		
**Age(years)**				2.348	0.503
< 30	36 (3.4)	17 (47.2)	19 (52.8)		
30–44	264 (25.3)	144 (54.5)	120 (45.5)		
45–59	381 (36.5)	186 (48.8)	195 (51.2)		
≥ 60	364 (34.8)	189 (51.9)	175 (48.1)		
**Place of residence**				18.717	0.000
Urban	966 (92.4)	477 (49.4)	489 (50.6)		
Rural	79 (7.6)	59 (74.7)	20 (25.3)		
**Level of education**				12.606	0.000
Junior high School or below	398 (38.1)	323 (58.3)	166 (41.7)		
Senior high School and above	647 (61.9)	304 (47.0)	343 (53.0)		
**Employment status**				2.303	0.129
Employed	622 (59.5)	307 (49.4)	315 (50.6)		
Others	423 (40.5)	229 (54.1)	194 (45.9)		
**Average monthly household income**^***a***^				30.904	0.000
1	242 (23.2)	144 (59.5)	98 (40.5)		
2	183 (17.5)	113 (61.7)	70 (38.3)		
3	227 (21.7)	117 (51.5)	110 (48.5)		
4	201 (19.2)	86 (42.8)	115 (57.2)		
5	192 (18.4)	76 (39.6)	116 (60.4)		
**Type of BMI**				14.363	0.000
UEBMI	547 (52.3)	250 (45.7)	297 (54.3)		
URRBMI	498 (47.7)	286 (57.4)	212 (42.6)		
**Medical expense burden**					
**Annual out-of-pocket medical expenses**				12.419	0.000
Low	630 (60.3)	351 (55.7)	279 (44.3)		
High	415 (39.7)	185 (44.6)	230 (55.4)		
**Out-of-pocket medical expenditure as a proportion of household non-food expenditure**				3.114	0.078
Low	522 (50.6)	240 (46.0)	282 (54.0)		
High	523 (49.4)	269 (51.4)	254 (48.6)		
**Health and disease status**					
**Health self-assessment**				0.669	0.183
Poor	576 (55.1)	284 (49.3)	292 (50.7)		
Good	469 (44.9)	225(48.0)	244 (52.0)		
**Chronic diseases**				1.073	0.300
No chronic diseases	859 (82.2)	447 (52.0)	412 (48.0)		
With chronic diseases	186 (17.8)	89 (47.8)	97 (52.2)		
**Cognition and information**					
**Familiarity with procedures of BMIS**				22.78	0.000
Low	720 (68.9)	405 (56.2)	315 (43.8)		
High	325 (31.1)	131 (40.3)	194 (59.7)		
**Accessibility of effective information about BMIS**				2.72	0.000
Low	620 (59.3)	355 (57.3)	265 (42.7)		
High	425 (40.6)	181 (42.6)	244 (57.4)		
**Understanding degree of effective information about BMIS**				55.049	0.000
Low	606 (52.0)	37 (61.6)	236 (38.9)		
High	439 (48.0)	166 (37.8)	273 (62.2)		
**Recent medical insurance use or affairs handling experiences**					
**Outpatient visits within two weeks**				17.773	0.000
No experience	990 (94.7)	523 (52.8)	467 (47.2)		
Have experience	55 (5.3)	13 (23.6)	42 (76.4)		
**Hospitalization within one year**				3.987	0.046
No experience	988 (94.5)	470 (47.6)	518 (52.4)		
Have experience	57 (5.5)	39 (68.4)	18 (31.6)		
**Daily drug purchase within one year**				9.377	0.002
No experience	189 (18.1)	114 (60.3)	75 (39.7)		
Have experience	856 (81.9)	422 (49.3)	434 (59.7)		
**Off-site medical treatment within one year**				1.296	0.255
No experience	1037 (99.2)	534 (51.5)	503 (48.5)		
Have experience	8 (0.8)	2 (25.0)	6 (75.0)		
**Visits to basic medical insurance institutions for business within one year**				0.001	0.976
No experience	957 (91.6)	491 (51.3)	466 (48.7)		
Have experience	88 (8.4)	45 (51.1)	43 (48.9)		

^a^Quintile 1 is the poorest and quintile 5 the wealthiest

### Factors associated with PCBMI

In the logistic regression model, four variables were significantly associated with PCBMI (*P* < 0.05). Those who have not had outpatient experience within two weeks (OR = 2.522, 95% CI = 1.267–5.024), have poor understanding of basic medical insurance information (OR = 2.336, 95% CI = 1.612–3.386), live in rural areas (OR = 1.819, 95% CI = 1.036–3.195), and have low annual out-of-pocket medical expenses (OR = 1.488, 95% CI = 1.129–1.961) are more likely to have poor PCBMI. Table 2 presents the details.

**Table 2:** Logistic regression analysis on the poor PCBMI

Variable	Walds	*P*-value	OR	95% CI	
**Social demography**					
** Place of residence**					
Rural	4.335	0.037	1.819	1.036	3.195
Urban (reference)					
**Level of education**					
Junior high School and below	1.209	0.272	1.170	0.884	1.550
Senior high School and above (reference)					
**Average monthly household income **^***a***^	3.834	0.429			
1	1.281	0.258	1.269	0.840	1.915
2	0.004	0.952	0.988	0.667	1.464
3	0.512	0.474	0.857	0.561	1.308
4	0.547	0.459	0.846	0.544	1.316
5 (reference)					
**Type of BMI**					
URRBMI	1.899	0.168	1.206	0.924	1.576
UEBMI (reference)					
**Medical expense burden**					
**Annual out-of-pocket medical expenses**					
Low	7.951	0.005	1.488	1.129	1.961
High (reference)					
**Cognition and information**					
**Familiarity with procedures of BMI**					
Low	0.102	0.749	1.060	0.741	1.516
High (reference)					
**The accessibility of effective information on BMI**					
Low	0.429	0.512	0.889	0.624	1.265
High (reference)					
**Understanding degree of effective information about BMIS**					
Low	20.094	0.000	2.336	1.612	3.386
High (reference)					
**Recent medical insurance use or affairs handling experiences**					
**Outpatient visits within two weeks**					
No experience	6.928	0.008	2.522	1.267	5.024
Have experience (reference)					
**Hospitalization within one year**					
No experience	0.771	0.380	0.750	0.394	1.426
Have experience (reference)					
**Daily drug purchase within one year**					
No experience	1.136	0.287	1.205	0.855	1.697
Have experience (reference)					
**Constants**	16.151	0.000	0.180		

^a^Quintile 1 is the poorest and quintile 5 the wealthiest

### Key problems with PCBMI

**Fig. 2:**
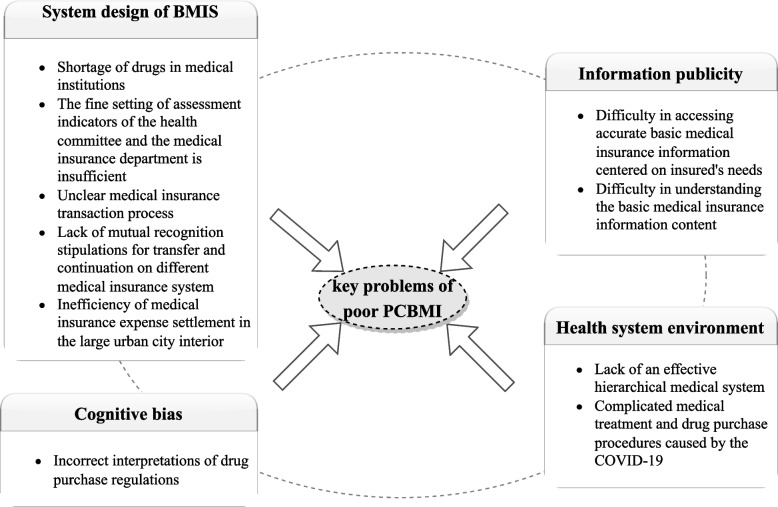
Qualitative analysis framework of insured’ poor PCBMI

Qualitative interviews mainly diagnose the details of specific problems of poor PCBMI, which are divided into four themes and related subthemes (Fig. 2). To promote understanding, we included quotations in the appendices (Supplementary file 2).

## Discussion

Improving convenience is the embodiment of the development of the BMIS towards better quality and service efficiency, especially as China makes progress with its universal health insurance coverage [36]. Our research provides information on the convenience of the BMIS in the process of deepening the reform of basic medical insurance in China from the perspective of the subjective perception of the insured. The quantitative research results show that the insured with the characteristics of lack of recent basic medical insurance use experience, inadequate understanding of basic medical insurance information, living in rural areas, and having low annual out-of-pocket medical expenses have poorer PCBMI. We need to pay more attention to those kinds of insured. Through in-depth interviews, we found that the main problems influencing PCBMI are concentrated in four aspects: the design of the BMIS, cognitive bias of the insured, BMIS information publicity, and the health system environment.

### Characteristics of insured with poor PCBMI

The insured with poor PCBMI have the following four characteristics: First, we found that those with a lack of recent basic medical insurance use experience had an obvious negative tendency to evaluate the convenience of BMIS. The possible explanation for this finding is that the public’s awareness of the improved policy reform is insufficient, especially in the medical service areas they do not frequently use [26]. Those who lack experience might precipitate the difficulty of obtaining medical service and inconvenient basic medical insurance in the past into the collective memory [37, 38]. Although China has made great efforts in recent years to improve the rationality of medical resource allocation, develop a hierarchical medical treatment system, establish an information system for basic medical insurance, and promote the convenience of basic medical insurance reimbursement [4, 26, 39, 40], due to the lack of personal experience, it is hard to update the negative collective memory compared with those who have recent basic medical insurance use experience. Therefore, poor evaluations of the PCBMI are inevitable. In future, on the basis of continuing to promote the improvement of the BMIS, there must be an increase in the insured’s awareness of real-time changes made to the BMIS and medical system.

In terms of specific basic medical insurance use experience, only those who have had outpatient experience within two weeks showed statistical significance in the recent basic medical insurance use experience dimension (OR = 2.522). This is probably mainly because compared to the insured with other experiences, those with outpatient experience are required to complete all basic medical insurance business in as short a time as possible. Therefore, they are more sensitive and have high expectations of the process and time investment of using basic medical insurance. However, many shortcomings in the convenience of basic medical insurances remain. The gap between expectation and reality easily reduces the PCBMI.

Second, poorer understanding of basic medical insurance information resulted in poorer PCBMI (OR = 2.336). The insured can obtain plenty of information from different sources, but this information is not always easily comprehended. According to Li et al., many insured have a poor understanding of the policy documents of basic medical insurance which inconvenienced them in the process of applying for basic medical insurance benefits [41]. The qualitative results of this study also confirmed this point. To improve the PCBMI, more focus should be given to groups with poor understanding of basic medical insurance information.

Third, the rural insured’s PCBMI are worse than that of the urban insured (OR = 1.819). Our qualitative results support those of previous studies that have confirmed that rural areas are more disadvantaged in terms of the efficiency of the medical system. For instance, the government invests less in the rural medical system and the rural information system is inadequate. Rural areas are also far away from high-quality medical institutions [42, 45].

Fourth, our findings showed that insured with low annual out-of-pocket medical expenses have worse PCBMI (OR = 1.488). Few studies have provided evidence of the relationship between annual out-of-pocket medical expenses and the convenience of BMIS. In general, insured persons with low annual out-of-pocket medical expenses have relatively mild illnesses, therefore they have a high expectation of investing in other factors besides the diagnosis and treatment [45], for example, the convenience of basic medical insurance and medical services. When basic medical insurance is not convenient to use, the PCBMI will be significantly reduced.

### Key problems influencing poor PCBMI

### System design of BMIS

Like other countries, China regards the establishment of a convenient basic medical insurance system a constant priority [10, 46]. However, this study showed that the PCBMI of the insured still requires improvement. The interviews mainly reflect that the design of the BMIS is imperfect in terms of the following aspects:

#### Shortage of drugs in medical institutions

The shortage of drugs in hospitals reduced the PCBMI. The lack of guarantee of the supply of drugs in Chinese hospitals, mainly drugs with low prices, occurs relatively frequently. A survey in 2016 based on 614 hospitals in China showed that the average occurrence rate of drugs shortage was 51 times per year, and low-price drugs account for 90% [47]. Additionally, the supply of drugs that are clinically necessary and used for emergencies, and drugs included in the national essential medicines list, is also insufficient to varying degrees [48]. Medical institutions in Harbin also face shortages [49]. The insufficient supply of drugs is a systematic and complex problem affected by factors such as price control, market demand, production costs, drug transportation, hospital management, and doctors’ habits [50]. Price policy control is the most important influencing factor [7]. In drug production, China's centralized drug procurement policy typically follows a "low price wins" strategy. However, China has less control over the price of raw materials used in drugs manufacturing and requires manufacturers to maintain the high standards of drug quality. Consequently, manufacturers face the potential risk of production costs exceeding profits, which may result in reduced motivation to continue production [51, 52], and the production companies may even cut the production of low-profit drugs. Furthermore, the varieties of selected drugs in centralized procurement are considerably less compared to those in the basic medical insurance catalog [53], therefore the price of these drugs is high. Preventing drug shortages in medical institutions is a prominent problem in the current drug supply reform, which guarantees ample supply. China should continue to improve the drug supply system to reduce the inconvenience of obtaining drugs.

#### The fine setting of assessment indicators of the health committees and the medical insurance departments is insufficient

Our study found that some insured complained that the hospital had decomposed hospitalization behavior, that is, patients’ hospitalization is divided into two or more stays. This is consistent with the reality in many Chinese cities, including Harbin [54, 55], which greatly affects the PCBMI of insured. Relevant research points out that this is highly related to the hospital needing to avoid the assessment indicators of the health commission and medical insurance department. Regarding the health committees, they assess hospital performance by setting indicators such as the average increase in hospitalization expenses, average increase in drug expenses during hospitalization, and the duration of hospitalization [56]. Therefore, for cases with high treatment costs, long treatment cycles, and complex complications, some hospitals require them to be discharged and hospitalized repeatedly to meet the indicator requirements. Regarding the medical insurance departments, they restrict doctors’ diagnosis and treatment behavior to control medical expenses through the diagnosis-related group payment method (DRG) [57]. However, the weight setting of the disease group and the types of diseases included in the disease group in the DRG payment method are imperfect. In the face of high cost patients, the hospitals have the risk of bearing the medical expenses beyond the basic medical insurance payment standard of patients, which also stimulates the hospitals to conduct decomposed hospitalization [58, 59]. It is gratifying that China is reforming the accuracy of indicators for assessing hospital performance, which may be improved in future.

#### Unclear basic medical insurance transaction process

The unclear business process of basic medical insurance management departments is another concern of the interviewees. The unclear service process is the result of the lack of standardization across management departments [60] and the lack of collaborative services with relevant service institutions. Low service efficiency forces the insured who are unfamiliar with the basic medical insurance business to go back and forth between departments, reducing their PCBMI. Therefore, the basic medical insurance management departments need to form a clear business process and realize the integration of basic medical insurance handling to meet the practical needs of the majority of insured.

#### Lack of mutual recognition stipulations for transfer and continuation on different medical insurance systems

The interviews revealed that the lack of mutual recognition stipulations between different BMIS during the transfer and continuation is also an obstacle affecting PCBMI (this study mainly refers to the transfer and continuation of urban and rural resident basic medical insurance (URRBMI) to the urban resident basic medical insurance (UEBMI) in the same region). Due to the design differences between the two medical insurance systems, such as differences in the fixed number of financing and payment, it is difficult for China to formulate specific stipulations on mutual recognition of payment years. The results of a survey involving 31 provincial capitals and municipalities showed that only a few regions adopt different methods to convert payment years. There are still many regions, including Harbin, that do not recognize the transfer and continuation of payment years from the URRBMI to UEBMI [15]. The poor transfer and continuity of insurance entitlements run counter to the continuous use of insurance by the insured, and requires additional attention.

#### Inefficiency of basic medical insurance expense settlement in the large urban city interior

The study also found that inefficiency in basic medical insurance expense settlement within the same urban cities reduces the insured’s PCBMI, which echoes the quantitative results showing the lower PCMBI of the rural insured. Harbin is a typical city which covers an area of over 53,000 square kilometers, and the distribution of medical resources is uneven with more resources in the city center than at the edge of the city [44]. Many residents have to travel long distances to reach hospitals. As hospitals and medical insurance agencies in Harbin currently reconcile medical expenses manually, auditing becomes slow. The insured patient must therefore return to the hospital again after a period of time to settle their medical expenses, which increases inconvenience. Repeated long journeys and icy roads during winter make the process more difficult for the insured, especially for complex basic medical insurance transactions that cannot be fulfilled at one time.

### Cognitive bias

People’s cognition is an important basis for decision-making and subjective judgment. However, in reality, it is prone to bias and causes people to make incorrect assessments [61]. This study found that there were cognitive biases among the insured, which are mainly reflected in the incorrect interpretation of drug purchase regulations.

#### Incorrect interpretations of drug purchase regulations

We found that the insured misinterpreted the drug purchase regulations. For example, a member was restricted by the number of drugs purchased by the retail drugstore, which led to her dissatisfaction and poor PCBMI. The original purpose of setting the limit on the number of one-time drug purchases is to prevent patients from abusing drugs without doctor permissions [62]. However, the rational drug use signal released by the restriction regulations on the number of drugs purchased is not effectively received by the insured. According to the research of Gu et al., the knowledge, attitude, and behavior of residents in Heilongjiang Province on rational drug use require improvement [63]. Contrary to the original intention of supporting the people covered by the policy, they tend to evaluate the regulations from the perspective of their own interests. This phenomenon reflects the deficiencies in basic medical insurance policy publicity in China, which confirms the speculation of this study.

### Information publicity

Basic medical insurance policy publicity is an important basis for deepening the understanding of the insured and allowing them to conveniently use the basic medical insurance benefits. However, this study found that the current publicity effect of the basic medical insurance policy was poor, which reduced the insured’s PCBMI. These problems were mainly reflected in their difficulty in accurately accessing and understanding basic medical insurance information.

#### Difficulty in accessing accurate basic medical insurance information

In this study, we found that it was difficult for the insured to easily obtain basic medical insurance information centered on their needs. The barriers to information increased their difficulty in using the basic medical insurance, which was similar to Liu’s research results [23]. First, many communication theories emphasize the necessity of interaction between information disseminators and recipients [64, 65]. However, the current insured is passive in the process of basic medical insurance information publicity. Massive basic medical insurance information is transmitted to the insured one-way through various media, and this information is usually not needed by the insured. Second, there are also obstacles for the insured to actively search for basic medical insurance information. For example, the insured does not know what channels are available for information searches [66]. The precision communication technology of information on many media platforms is insufficient [67], and the update of basic medical insurance information on media platforms is lacking. Third, the digital poor represented by the elderly, find it difficult to adapt to the current development of medical informatization. Many studies have shown that the digital information literacy of the elderly is low [68, 69], and they find it difficult to understand and operate information technology to obtain basic medical insurance information related to their own health. In summary, these problems have hindered their smooth use of basic medical insurance and reduced their PCBMI.

#### Difficulty in understanding basic medical insurance information content

Effective understanding of policy information enables people to take action according to policy requirements without hindrance. However, our study found that the insured had a poor understanding of basic medical insurance policy information which reduced their PCBMI. Previous studies have reached the same conclusion [46]. Medical insurance information is usually released by the authoritative medical insurance department. This information is usually provided in professional terms, which is difficult to comprehend by those with poor policy literacy. Some policy publicity practices of the China Medical Insurance Bureau have proved that the insured are more likely to accept a policy language that is easy to understand [70]. Based on this reality, China is making efforts to improve the expression of medical insurance policy language by, for example, using simple words and vivid pictures for better understanding. However, the perception of the insured on these improvement measures is still not clear and needs to be explored further.

### Health system environment

The qualitative research results showed that the health system environment was also an important problem causing poor PCBMI. We found that a halo effect was produced when the insured assessed PCBMI [71]. In other words, when the insured are affected by the perception that some elements in the system environment are inconvenient to use, their assessment of the convenience of basic medical insurance related to these elements is also indirectly affected. In this theme, the respondents were dissatisfied with the inconvenient operation of the hierarchical medical system and the cumbersome medical care and drug purchase procedures caused by COVID-19, which affected their evaluation of PCBMI.

#### Lack of an effective hierarchical medical system

This study found that the imperfect hierarchical medical system affected the insured’s experience of using medical services smoothly, which indirectly prompted them to have low PCBMI. Although the hierarchical medical system in Heilongjiang Province has gradually improved in recent years, which has eased the pressure on the insured’s difficulty in obtaining medical services to some extent, there are still some deficiencies [72]. For example, some primary health care institutions are not perfect in terms of doctor capacity, drug allocation, and equipment allocation [73, 75]. Moreover, differentiated reimbursement policies in the referral system have not been effectively implemented. Therefore, the insured still prefer to go directly to high-level hospitals. At present, some famous large hospitals in Harbin remain overcrowded [76], which reduces the convenience of medical services and their PCBMI.

#### Complicated medical treatment and drug purchasing procedures caused by COVID-19

According to the interview results, we found that the impact of COVID-19 prompted China to implement a series of emergency plans in various provinces and cities, effectively reducing the spread of the virus [77]. However, it also made the management procedures for obtaining medical treatment and purchasing daily drugs more complicated. For example, new measures such as online appointment registration, temperature measurement, scanning of health codes before treatment, and the provision of identity information when buying fever drugs at the pharmacy, have been implemented [78]. Although some of the insured agree with these measures, the cumbersome medical care and drug purchase procedures will still indirectly affect their daily use of basic medical insurance and increase their perceptions of inconvenience.

### Policy recommendations

Based on the results, the government should formulate policies that consider the needs of insured without recent basic medical insurance experience, have insufficient understanding of basic medical insurance information, live in rural areas, and have low annual out-of-pocket medical expenses. In addition, various strategies should be considered to address key problems and improve the convenience of the BMIS.

Regarding the BMIS design, first, the government should improve the drug supply guarantee mechanism, and strengthen the management of drug price formulation, purchase volume, and distribution. Each city should establish an early warning mechanism for drug shortages. Second, the medical insurance department and the health committee should optimize the refined management of BMIS payment methods, and increase the scientificity and rationality of the assessment indicators according to the service characteristics of medical institutions. Third, the medical insurance bureaus of various cities and regions should formulate clear basic medical insurance service handling procedures. The government should oversee the coordination and cooperation of the relevant institutions handling medical insurance transactions. Fourth, the stipulations on the transfer and continuation of the cross medical insurance system should be formulated as soon as possible to meet the needs of the vast numbers of insured. Fifth, the government needs to increase investment in medical resources in the surrounding areas of the city. It needs to build and improve the information system construction of medical institutions and medical insurance agencies, focusing on intelligent audits assisted by manual services, to enhance the efficiency of basic medical insurance expense settlements.

Regarding the cognitive bias of insured and BMIS information publicity, publicity channels, content, and form, as well as collection channels, should be improved to better assist insured with their concerns about basic medical insurance. The government and media platforms should also improve the communication effect of basic medical insurance information based on information technology by adopting the scenario publicity model and improving the precision retrieval technology of basic medical insurance information on the internet. Moreover, the information output department should pay attention to the use of policy language that is easy for the public to understand and give thorough explanations of the information in the process of publicity. Furthermore, it should strengthen digital information literacy education for the digital poor.

Regarding the health system environment, the government needs to improve the overall performance of the system by, for example, improving the hierarchical medical system and optimizing the overall measures to deal with major epidemics.

## Conclusions

This study used a mixed research method to explore the characteristics, influencing factors, and key barriers of the insured with low PCBMI. Different from previous studies, we found that in addition to the design of the BMIS, the cognition of the insured, the BMIS information publicity and the health system environment are also the key problems hindering PCBMI. Therefore, to improve the PCBMI of the insured, Chinese policy makers need to pay attention to the insured with low PCBMI characteristics while optimizing the system design and implementation. Moreover, it is necessary to focus on exploring effective BMIS information publicity methods, supporting public policy literacy, and improving the health system environment.

## Limitations

This study had several limitations. First, in the quantitative, due to the effect of the COVID-19, the respondents with recent basic medical insurance use experience was low and also affected the subjective evaluation of respondents to some extent. Second, owing to resource and condition constraints, only the insured in Harbin were investigated, therefore the survey results can only represent the situation of Harbin and the regions similar to it, and cannot be extrapolated to the entire country. To reflect the situation in China as a whole, further research involving a wider range of regions and populations are necessary. Despite recognizing these limitations, we believe that the results and findings of this study could provide a reference for other areas where the convenience of basic medical insurance is undergoing reform.

## Supplementary Information

**Additional file 1.**

**Additional file 2.**

**Additional file 3: Supplementary file 1. **Logistic regression analysison the poor PCBMI.

**Additional file 4: Supplementary file 2. **Qualitative Research Findings.

## Data Availability

The datasets used and/or analysed during the current study are available from the corresponding author on reasonable request.

## Abbreviations

BMIS: Basic medical insurance system
PCBMI: Perceptions of convenience of the basic medical insurance
UEBMI: Urban Resident Basic Medical Insurance
URRBMI: Urban and Rural Resident Basic Medical Insurance
