# Supplementary material for: Factors and key problems influencing insured’s poor perceptions of convenience of basic medical insurance: a mixed methods research of a northern city in China
Source: BMC Public Health. 2023 Jun 5;23:1066. doi: 10.1186/s12889-023-15993-1 (PMC10240473; doi:10.1186/s12889-023-15993-1)
Supplement: Supplementary file 1 — Additional file 1. [file 12889_2023_15993_MOESM1_ESM.docx]

**An interview outline of** **the** **perceptions of convenience of basic medical insurance**

Dear Sir/Madam,

We are the research team from Harbin Medical University who are investigating “The perceptions of convenience of basic medical insurance of the insured." The main purpose of this interview is to understand the practical problems and your perceptions related to the convenience of basic medical insurance.

Our interview will be 30 – 60 minutes by telephone which will be recorded. We guarantee that the data obtained will be used only for academic research and your personal data will not be disclosed.

If you are willing to participate in our interview, please send your valid contact information to the project contact person (please indicate that you have agreed to the interview). We will pay a consultation fee of 50 yuan to thank you for your time and effort.

The telephone interview will be comprised of the six questions below. Please read and think about them in advance so that the interview proceeds smoothly. Thank you very much for your cooperation and time.

1. How old are you?

2. What is your education level?

3. Where do you live, in a rural or urban area?

4. What is your employment status?

5. What is your current income?

6. Please refer to (but do not be limited by) the content in the following table and combine your basic medical insurance use experience to share the three aspects related to the convenience of basic medical insurance that you are most dissatisfied with. Please provide details.

| Perceptions of convenience of basic medical insurance of the insured | How cumbersome is the "participation" process? How time-consuming is it? |
| --- | --- |
|  | How cumbersome is the process of "medical insurance relationship transfer and connection"? How time-consuming is it? |
|  | How cumbersome is the Medicare "reimbursement process?" How time-consuming is it? |
|  | How reasonable is the "geographical distribution" of designated medical insurance institutions and designated pharmacies? |
|  | How easy is it to use basic medical insurance to buy drugs? |
|  | In the context of the COVID-19 epidemic, has the convenience of basic medical insurance changed and if so, what has changed? What is inconvenient about having treatment or buying medicine? |
