# Supplementary material for: Factors and key problems influencing insured’s poor perceptions of convenience of basic medical insurance: a mixed methods research of a northern city in China
Source: BMC Public Health. 2023 Jun 5;23:1066. doi: 10.1186/s12889-023-15993-1 (PMC10240473; doi:10.1186/s12889-023-15993-1)
Supplement: Supplementary file 2 — Additional file 2. [file 12889_2023_15993_MOESM2_ESM.docx]

**Questionnaire of the perceptions of convenience of basic medical insurance**

**Informed consent**

Dear Madam/Sir,

Welcome, and thank you for participating in this survey. We are the research team from Harbin Medical University who are investigating “The perceptions of convenience of basic medical insurance of the insured." The main purpose of designing and conducting this questionnaire is to understand the real perceptions of convenience of the insured regarding basic medical insurance, objectively evaluate the actual implementation effect of convenience of the current basic medical insurance system, and provide suggestions for its improvement.

To ensure the objectivity and privacy of the survey content, we will use codes instead of names to ensure that your answers are strictly confidential. The survey data obtained will only be used for the research and will only be visible to the researchers. If you answer and submit our questionnaire, we will consider that you understand and agree to accept the survey. Next, please answer each question truthfully according to your actual views and perceptions of the basic medical insurance system.

Thank you for your cooperation!

Questionnaire code:

1. Have you have participated in the basic medical insurance: ① Yes ② no

(This questionnaire only investigates the personnel who have participated in basic medical insurance.)

**I. Demographic and socioeconomic characteristics**

1. Sex：①Female ②Male

2. Your birth year is ______

3. Place of residence：①Rural ②Urban

4. Your highest level of education：

①Junior high School or below

②Senior high School and above

1. Employment status

①Employed

②Others

1. The average monthly income of your family is approximately ______ CNY/month

7. Type of basic medical insurance：

①UEBMI

②URRBMI

**II. Health and disease status**

1. Out of 100, how would you rate your current health status? ________

2. The top three major chronic diseases you have been diagnosed with are:

2.1 The "first" main chronic diseases: ① hypertension ② diabetes ③ cardiovascular and cerebrovascular diseases ④ others: ___ ⑤ none (If your chronic disease does not appear in the above options, please fill in the specific name of your chronic disease elsewhere)

2.2 The "second" main chronic disease name: ① hypertension ② diabetes ③ cardiovascular and cerebrovascular diseases ④ others: ___ (If your chronic disease does not appear in the above options, please fill in the specific name of your chronic disease elsewhere) ⑤ none

2.3 The "third" main chronic disease name: ① hypertension ② diabetes ③ cardiovascular and cerebrovascular diseases ④ others: ___ (If your chronic disease does not appear in the above options, please fill in the specific name of your chronic disease elsewhere) ⑤ none

Note: Chronic diseases refer to those who meet one of the following conditions:

1. Chronic diseases clearly diagnosed by medical personnel within six months before the survey;

2. The patient had a chronic disease diagnosed by a doctor half a year ago, and had had an attack from time to time during the first half a year and had taken treatment measures, such as taking medicine, physiotherapy, or been receiving treatment to control the attack of the chronic disease, etc.

**III. Medical expense burden**

1. Your family's out-of-pocket medical expenses (including prevention, health care, rehabilitation, treatment and other services) in the previous year were about _____ CNY (excluding the part reimbursed by basic medical insurance and the expenditure of basic medical insurance personal accounts).

2. Your family's non-food expenses in the previous year were about _____ CNY.

**IV. Mastery and accessibility of basic medical insurance information**

1. Do you agree that you are familiar with basic medical insurance procedures and information?

①completely disagree ②disagree ③neither disagree nor agree

④agree ⑤completely agree

2. Do you agree that the basic medical insurance procedures and information are easily accessible to you?

① completely disagree ② disagree ③ neither disagree nor agree

④ agree ⑤ completely agree

3. Do you agree that you have a good understanding of basic medical insurance procedures and information?

① completely disagree ② disagree ③ neither disagree nor agree

④ agree ⑤ completely agree

**V. Basic medical insurance use experience**

1. Have you experienced outpatient visits within two weeks?

① have experience ② no experience

1.1 Did you use basic medical insurance for reimbursement for this experience?

① yes ② no

1.2 For the medical treatment, the reason you did not reimburse was (optional) :

① Diagnosis and treatment items occurring are not in the scope of basic medical insurance ② The procedures related to reimbursement are too tedious and troublesome ③ The cost of the illness is low and does not need reimbursement ④ Poor geographical access to designated medical institutions ⑤ Skip

1.3 If you did not have treatment, was it because of economic reasons?

① a large degree ② relatively large degree ③ general

④ a small degree ⑤ completely

2. Have you experienced hospitalization within one year?

① have experience ② no experience

2.1 Have you had any medical diagnoses requiring hospitalization that you have not had in the last year?

① yes ② no

2.2 For the medical treatment, the reason you did not reimburse was (optional) :

① Diagnosis and treatment items occurring are not in the scope of basic medical insurance ② The procedures related to reimbursement are too tedious and troublesome ③ The cost of the illness is low and does not need reimbursement ④ Poor geographical access to designated medical institutions ⑤ Skip

2.3 If so, to what extent is it for economic reasons?

① a large degree ② relatively large degree ③ general

④ a small degree ⑤ completely

3. Have you experienced daily drug purchases within one year?

① have experience ②no experience

4. Have you experienced receiving off-site medical treatment within one year?

① have experience ②no experience

5. Have you experienced visiting medical insurance institutions for business within one year?

①have experience ②no experience

Note: The medical insurance related matters to be handled include: ① to apply for basic medical insurance ② to apply for medical insurance relationship transfer and renewal ③ to apply for chronic disease medical insurance ④ to apply for off-site medical ⑤ to apply for serious disease medical insurance ⑥ to change the password of medical insurance accounts ⑦ to query the current medical insurance account

**VI. Evaluation form of the insured's PCBMI**

Comments: Please tick "√" under the following questions according to how much you agree with them.

| **Item** | **completely disagree** | **disagree** | **neither disagree nor agree** | **agree** | **completely agree** |
| --- | --- | --- | --- | --- | --- |
| 1. In general, I think the current procedures for participating in basic medical insurance are very convenient. |  |  |  |  |  |
| 2. In general, I think the current procedures for payment of basic medical insurance premiums are very convenient. |  |  |  |  |  |
| 3. In general, I think the current procedures for the transfer and connection of basic medical insurance are very convenient (for example, the process of the transfer and connection of the medical insurance relationship between UEBMI and URRBMI includes a series of processes such as the change in medical insurance relationship and the continuation of the medical insurance account expenses and payment period after the change). |  |  |  |  |  |
| 4. In general, I think the current procedures for outpatient and emergency reimbursement are very convenient. |  |  |  |  |  |
| 5. In general, I think the current procedures for hospitalization reimbursement are very convenient. |  |  |  |  |  |
| 1. In general, I think the current procedures for drug purchase reimbursement are very convenient. |  |  |  |  |  |
| 7. In general, I think the current procedures for off-site medical application are very convenient. |  |  |  |  |  |
| 8. In general, I think the current procedures for off-site medical reimbursement are very convenient. |  |  |  |  |  |
| 9. In general, I think the current geographic distribution convenience of designated medical institutions is reasonable (the insured can reach the designated medical institutions near their places of residence for medical treatment within 15 minutes). |  |  |  |  |  |
| 10. In general, I think the current geographic distribution convenience of designated pharmacies is reasonable (the insured can go to the designated medical insurance pharmacies near their places of residence to buy medicine within 15 minutes). |  |  |  |  |  |
