# Supplementary material for: Factors and key problems influencing insured’s poor perceptions of convenience of basic medical insurance: a mixed methods research of a northern city in China
Source: BMC Public Health. 2023 Jun 5;23:1066. doi: 10.1186/s12889-023-15993-1 (PMC10240473; doi:10.1186/s12889-023-15993-1)
Supplement: Supplementary file 3 — Additional file 3: Supplementary file 1. Logistic regression analysison the poor PCBMI. [file 12889_2023_15993_MOESM3_ESM.docx]

**Supplementary file 1. Logistic regression analysis on the poor PCBMI**

| **Variable** | **Walds** | ***P*-value** | **OR** | **95% CI** | |
| --- | --- | --- | --- | --- | --- |
| **Social demography** |  |  |  |  |  |
| **Sex** |  |  |  |  |  |
| Female | 3.397 | 0.065 | 1.288 | 0.984 | 1.686 |
| Male (reference) |  |  |  |  |  |
| **Age（years）** | 7.335 | 0.062 |  |  |  |
| <30 | 2.376 | 0.123 | 1.801 | 0.852 | 3.808 |
| 30-44 | 0.135 | 0.714 | 1.151 | 0.544 | 2.436 |
| 45-59 | 0.160 | 0.689 | 1.176 | 0.531 | 2.608 |
| ≥60 (reference) |  |  |  |  |  |
| **Place of residence** |  |  |  |  |  |
| Rural | 4.365 | 0.037 | 1.838 | 1.038 | 3.252 |
| Urban (reference) |  |  |  |  |  |
| **Level of education** |  |  |  |  |  |
| Junior high School and below | 1.482 | 0.223 | 1.209 | 0.891 | 1.642 |
| Senior high School and above (reference) |  |  |  |  |  |
| **Employment status** |  |  |  |  |  |
| Employed | 1.187 | 0.277 | 0.816 | 0.566 | 1.177 |
| Others (reference) |  |  |  |  |  |
| **Average monthly household income *^a^*** | 4.450 | 0.349 |  |  |  |
| 1 | 1.081 | 0.298 | 1.252 | 0.820 | 1.911 |
| 2 | 0.145 | 0.703 | 0.923 | 0.613 | 1.392 |
| 3 | 0.928 | 0.335 | 0.802 | 0.512 | 1.256 |
| 4 | 0.835 | 0.361 | 0.802 | 0.500 | 1.287 |
| 5 (reference) |  |  |  |  |  |
| **Type of BMI** |  |  |  |  |  |
| URRBMI | 1.146 | 0.284 | 1.164 | 0.882 | 1.536 |
| UEBMI (reference) |  |  |  |  |  |
| **Medical expense burden** |  |  |  |  |  |
| **Annual out-of-pocket medical expenses** |  |  |  |  |  |
| Low | 4.332 | 0.037 | 1.390 | 1.019 | 1.894 |
| High (reference) |  |  |  |  |  |
| **Out-of-pocket medical expenditure as a proportion of household non-food expenditure]** |  |  |  |  |  |
| Low | 1.808 | 0.179 | 0.748 | 0.490 | 1.142 |
| High (reference) |  |  |  |  |  |
| **Health and disease status** |  |  |  |  |  |
| **Health self-assessment** |  |  |  |  |  |
| Poor | 1.062 | 0.303 | 1.174 | 0.865 | 1.592 |
| Good (reference) |  |  |  |  |  |
| **Chronic diseases** |  |  |  |  |  |
| No chronic diseases | 1.351 | 0.245 | 1.277 | 0.845 | 1.929 |
| With chronic diseases (reference) |  |  |  |  |  |
| **Cognition and information** |  |  |  |  |  |
| **Familiarity with procedures of BMI** |  |  |  |  |  |
| Low | 0.161 | 0.688 | 1.079 | 0.745 | 1.562 |
| High (reference) |  |  |  |  |  |
| **The accessibility of effective information on BMI** |  |  |  |  |  |
| Low | 0.060 | 0.806 | 0.956 | 0.665 | 1.372 |
| High (reference) |  |  |  |  |  |
| **Understanding degree of effective information about BMIS** |  |  |  |  |  |
| Low | 19.427 | 0.000 | 2.330 | 1.600 | 3.394 |
| High (reference) |  |  |  |  |  |
| **Recent medical insurance use or affairs handling experiences** |  |  |  |  |  |
| **Outpatient visits within two weeks** |  |  |  |  |  |
| No experience | 9.247 | 0.002 | 3.169 | 1.507 | 6.664 |
| Have experience (reference) |  |  |  |  |  |
| **Hospitalization within one year** |  |  |  |  |  |
| No experience | 1.167 | 0.280 | 0.691 | 0.354 | 1.351 |
| Have experience (reference) |  |  |  |  |  |
| **Daily drug purchase within one year** |  |  |  |  |  |
| No experience | 0.815 | 0.367 | 1.178 | 0.826 | 1.679 |
| Have experience (reference) |  |  |  |  |  |
| **Off-site medical treatment within one year** |  |  |  |  |  |
| No experience | 1.177 | 0.278 | 2.552 | 0.470 | 13.864 |
| Have experience (reference) |  |  |  |  |  |
| **Visits to basic medical insurance institutions for business within one year** |  |  |  |  |  |
| No experience | 1.762 | 0.184 | 1.398 | 0.853 | 2.291 |
| Have experience (reference) |  |  |  |  |  |
| **Constants** | 9.760 | 0.002 | 0.036 |  |  |

***a Quintile 1 is the poorest and quintile 5 the wealthiest***

***All variables are included in this model***
