# Supplementary material for: Factors and key problems influencing insured’s poor perceptions of convenience of basic medical insurance: a mixed methods research of a northern city in China
Source: BMC Public Health. 2023 Jun 5;23:1066. doi: 10.1186/s12889-023-15993-1 (PMC10240473; doi:10.1186/s12889-023-15993-1)
Supplement: Supplementary file 4 — Additional file 4: Supplementary file 2. Qualitative Research Findings. [file 12889_2023_15993_MOESM4_ESM.docx]

**Supplementary file 2. Qualitative Research Findings**

| **Advanced theme** | **Primary theme** | **Illustrative quotes from enrollee interviews** |
| --- | --- | --- |
| System design of BMIS | Shortage of drugs in medical institutions | *I have to go outside pharmacies to buy some general medicines since they are not available at most hospitals. (Material 2)* |
|  | The fine setting of assessment indicators of the health committee and the medical insurance departments is insufficient | *The hospital will limit our length of stay to about one week before discharging us. If the patient’s condition is slightly worse, hospitals will force them to be discharged within half a month. This phenomenon is very common among the patients around me. (Material 3)*  *Nowadays, hospitals only let patients stay for half a month at most when they seem well but are not yet completely cured. My mother had the same experience when she was hospitalized. (Material 23)* |
|  | Unclear medical insurance transaction process | *I encountered many obstacles when applying for a medical insurance card for my 5-year-old nephew because the whole process is very unclear. Many department staff I consulted advised me that their department is not responsible for handling my concern. In the end, it took me two days to go to five departments to help me with my situation. (Material 22)* |
|  | Lack of mutual recognition stipulations for transfer and continuation on different medical insurance system | *I enrolled in the URRBMI, but when I changed to the UEBMI, I found that the premium years paid previously could not be transferred or converted to the premium years of UEBMI. (Material 28)* |
|  | Inefficiency of medical insurance expense settlement in the large urban city interior | *We come from the countryside. Even if we are sick, if we are not particularly uncomfortable, we will not go to the hospital because we live far away from large hospitals. (Material 12)*  *I live in the suburb of Harbin (Shuangcheng). There is no good hospital here. When I get sick, I need to go to the big hospital in Harbin but the road from Shuangcheng to Harbin is very inconvenient. During the winter, it takes about two or three hours to get there. (Material 18)* |
| Cognitive bias | Incorrect interpretations of drug purchase regulations | *I need to eat Metformin hydrochloride tablets regularly because of diabetes, so I want to buy 10 boxes at one time. However, the pharmacy staff advised me that I could only buy five boxes at most. If I want to buy 10 boxes, I can just buy five today and another five tomorrow. This is clearly teasing people. Isn't it more convenient to buy 10 boxes of medicine at one time? (Material 3)* |
| Information publicity | Difficulty in accessing accurate basic medical insurance information | *I learn some medical insurance information at ordinary times, but this information has little connection with my own disease. I usually obtain the medical insurance information I need through my own medical experience, which takes much time and energy. (Material 3)*  *I have a need to know medical insurance information, such as the reimbursement amount for hospitalization, which drugs and examinations are covered by subsidies, and so on. Although these pieces of information are public, they do not usually get my attention. When I need it, I can’t find the information I want in a short time. (Material 20)*  *Nowadays, information technology is so common in society, and it is very difficult for the elderly to use it. We do not know how to collect medical and basic medical insurance information, and we need to rely on young people. (Material 30)* |
|  | Difficulty in understanding basic medical insurance information content | *Some medical insurance policy information is difficult to understand. For example, some of the medical insurance related policy information mentioned in the news are very difficult for me to understand because of the jargon or technical language. (Material 22)* |
| Health system environment | Lack of an effective hierarchical medical system | *Generally, I do not go to primary medical institutions. I still prefer a larger hospital because it has better equipment and doctors. (Material 22)*  *Because of some bad medical experiences I’ve had before, I don’t trust primary medical institutions anymore. (Material 20)* |
|  | Complicated medical treatment and drug purchasing procedures caused by the COVID-19 | *During the epidemic, hospital appointments could only be made through online registration, and the number of allotted appointments is fewer. If you don’t make an appointment, you can’t receive medical treatment. These procedures are very troublesome. (Material 11)*  *During the COVID-19 epidemic, it is particularly difficult to buy fever drugs in pharmacies. Pharmacies need to register ID information, scan health codes, and register mobile phone numbers. While these procedures are troublesome, they can help with the prevention and control of the epidemic. However, because these procedures are complicated, I choose not go to the pharmacy to buy medicines, unless my illness is very serious. (Material 5)*  *The procedures for getting medical treatment and buying medicine are very complicated during the epidemic. However, even if one has to go through many steps with these procedures and people need to queue up for a long time, I can tolerate such procedures since they are in place to control the spread of the virus. (Material 7)* |
